# Tetrodotoxin Concentrations in *Pleurobranchaea maculata*: Temporal, Spatial and Individual Variability from New Zealand Populations

**DOI:** 10.3390/md10010163

**Published:** 2012-01-17

**Authors:** Susanna A. Wood, David I. Taylor, Paul McNabb, Jarrod Walker, Janet Adamson, Stephen Craig Cary

**Affiliations:** 1 Cawthron Institute, Private Bag 2, Nelson 7042, New Zealand; Email: dave.taylor@cawthron.org.nz (D.I.T.); paul.mcnabb@cawthron.org.nz (P.M.); janet.adamson@cawthron.org.nz (J.A.); 2 Department of Biological Sciences, University of Waikato, Private Bag 3105, Hamilton 3240, New Zealand; Email: c.cary@waikato.ac.nz; 3 Department of Chemistry, Otago University, P.O. Box 56, Dunedin 9054, New Zealand; 4 Auckland Council, 1 The Strand, Level 4, Takapuna, Auckland 0622, New Zealand; Email: jarrod.walker@aucklandcouncil.govt.nz

**Keywords:** tetrodotoxin, *Pleurobranchaea maculata*, geographic variability, temporal variability

## Abstract

Tetrodotoxin (TTX) is a potent neurotoxin that has been identified in a range of phylogenetically unrelated marine and terrestrial organisms. Tetrodotoxin was recently detected in New Zealand in *Pleurobranchaea maculata* (the grey side-gilled sea slug). From June 2010 to June 2011 wild specimens were collected from 10 locations around New Zealand. At one site (Narrow Neck Beach, Auckland) up to 10 individuals were collected monthly for 6 months. Attempts were also made to rear *P. maculata* in captivity. Tetrodotoxin was detected in samples from eight of the ten sites. The highest average (368.7 mg kg^−1^) and maximum (1414.0 mg kg^−1^) concentrations were measured in samples from Illiomama Rock (Auckland). Of the toxic populations tested there was significant variability in TTX concentrations among individuals, with the highest difference (62 fold) measured at Illiomama Rock. Tetrodotoxin concentrations in samples from Narrow Neck Beach varied temporally, ranging from an average of 184 mg kg^−1^ in June 2010 to 17.5 mg kg^−1^ by December 2010. There was no correlation between TTX levels and mass. The highest levels correspond with the egg laying season (June–August) and this, in concert with the detection of high levels of TTX in eggs and early larval stages, suggests that TTX may have a defensive function in *P. maculata*. Only one larva was successfully reared to full maturation and no TTX was detected.

## 1. Introduction

Tetrodotoxin (TTX) is a non-protein, low molecular weight neurotoxin. It is highly potent when ingested, binding to sodium channels and interfering with muscle and nerve function [1]. TTX was long believed to be present only in pufferfish of the family Tetraodontidae, however, since its detection in the eggs of the California newt (*Taricha torosa*) in 1964 [2], TTX has been detected in a wide range of phylogenetically unrelated terrestrial and aquatic taxa including; one dinoflagellete, red calcareous algae, arthropods, echinoderms, molluscs, worms, newts and frogs [3]. Despite many decades of research there is uncertainty surrounding the biological origin of TTX, and its ecological function in TTX-containing organisms.

Many researchers have postulated that bacteria are responsible for producing TTX and multiple TTX-producing bacteria have been isolated from marine organisms [4,5]. However, the TTX concentrations produced by these bacteria are too low to account for the levels found in toxic organisms [3]. This, in concert with a lack of controls, non-specific techniques and cross-reactivity problems with the various methods used to detect TTX have introduced doubt around TTX’s microbial origins [6,7,8]. Evidence supporting endogenous production of TTX has been derived predominately from terrestrial taxa [9,10,11,12]. In captivity, TTX levels in the terrestrial newt *T. granulosa* increase with a TTX-free diet, and when induced to release TTX from their skin by electrical stimulation, regeneration of TTX occurs within nine months [11]. Additionally, Lehman *et al.*, [13] found no evidence of bacterial symbionts (mtDNA signatures) in TTX-laden organs of *T. granulosa*. 

Regardless of its origin, there are likely physiological costs to the TTX-conatining organisms associated with harbouring, accumulating and maintaining TTX and/or its producers, and thus any benefits must outweigh these energetic expenditures. Research suggests multiple ecological roles for TTX, which differ among organisms. These include: a defensive agent, e.g., the eggs and skin of many TTX-containing organisms have high levels of the toxin [12], an offensive weapon e.g., venom [14], and within- and between-species communication about the location of mates and potential food sources [15,16]. Among well-studied TTX-containing species both toxic and non-toxic populations have been identified (e.g., puffer fish and newts [17,18]), confounding these hypotheses somewhat. In *T. granulosa* marked differences in TTX between populations have been explained through chemically mediated co-evolution with its prey, the common garter snake–*Thamnophis sirtalis* [19]. Despite TTX having been identified in at least six phyla of organisms [5] *T. granulosa* are the only animals that contain TTX for which a resistant predator is known [19]. 

In 2009, an investigation into a series of dog poisonings on beaches in Auckland (North Island, New Zealand), led to the identification of TTX in *Pleurobranchaea maculata* (grey side-gilled sea slug). *Pleurobranchaea* *maculata* are highly opportunistic carnivores that scavenge on a range of invertebrate organisms (sea anemones, marine worms, other molluscs [20]). They are hermaphrodites and are found in shallow subtidal areas around New Zealand, and have also been recorded in southeastern Australia, China, Sri Lanka and Japan [21]. There are several features of *P. maculata* that make them a very amenable marine organism for investigating the origin and ecological function of TTX. They are found in relatively confined, easily accessible, shallow sub-tidal areas, as opposed to many other TTX-containing marine organisms, and they can be reared in captivity under very controlled conditions [21].

**Figure 1:**
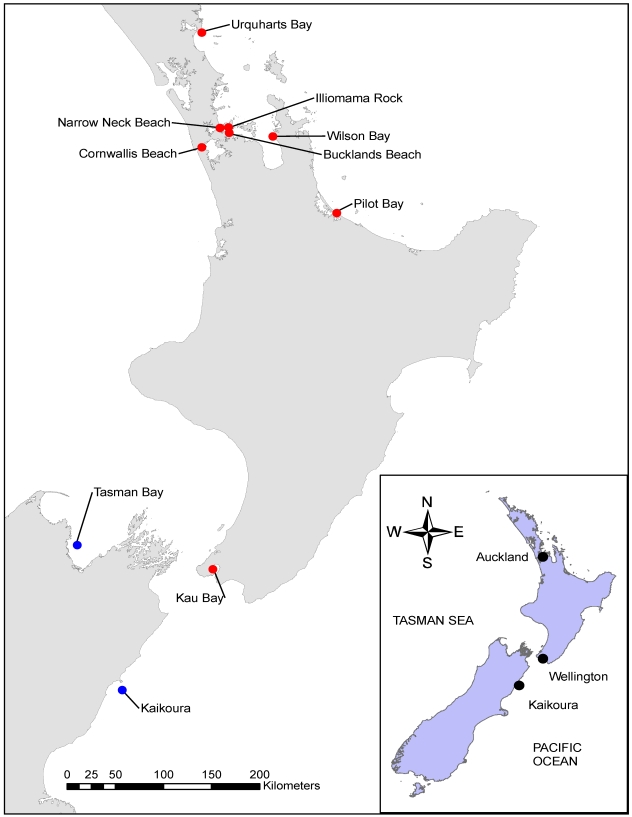
Sampling locations. Tetrodotoxin positive sites are plotted in red and negative in blue.

The aims of this study were to investigate temporal, spatial, individual and generational variability in TTX concentrations from New Zealand populations of *P.* *maculata*. Samples were collected from ten populations around New Zealand (Figure 1) and their TTX levels assessed using liquid chromatography-mass spectrometry (LC-MS). *Pleurobranchaea* *maculata* were also collected monthly over six months at Narrow Neck Beach (Auckland, New Zealand), to study temporal variability in TTX concentrations. To ensure differences in TTX (see results) were not due to the presence of cryptic species between North and South islands, we extracted DNA and sequenced a region of the cytochrome oxidase subunit 1 (CO1). Generational variability was examined by rearing *P.* *maculata* from eggs to maturation in a TTX-free environment. These data may provide insights into the origins and ecological function of TTX in *P.* *maculata.*

## 2. Results

### 2.1. Spatial Variability in Tetrodotoxin Concentrations in *Pleurobranchaea maculata* from Around New Zealand

Tetrodotoxin was detected in *P. maculata* samples from eight of the ten sites (Figure 1; Table 1). The highest average (368.7 mg kg^−1^) and maximum (1414.0 mg kg^−1^) concentrations were measured in samples from Illiomama Rock (Table 1). Traces levels (<0.2 mg kg^−1^) of 11-oxoTTX were detected in several *P. maculata* from Illiomama Rock. The lowest concentration of TTX (0.1 mg kg^−1^) was measured in the individual collected from Wilsons Bay (Figure 1; Table 1). At all sites where more than one individual was collected there was significant individual to individual variability, with a 7 (Pilot Bay) to 62 (Illiomama Rock) fold difference between the minimum and maximum TTX concentration (Table 1). Despite over 20 individuals been tested, no TTX was detected in the *P. maculata* from Tasman Bay. Likewise no TTX was detected in a single sample from the deep (290 m) site just off the Kaikoura coast. ANOVA analysis showed that the differences in TTX concentrations among populations of *P. maculata* were statistically significant (*F* = 34.2218, *df* = 5, *p* ≤ 0.0001). Pair-wise comparisons showed which populations were statistically different from each other (Table 2). The lower TTX concentrations in the Buckland Beach samples may be an artifact of their November collection date. The other toxic populations were sampled between June to August (see section below for data on temporal variation in TTX concentrations). 

**Table 1:** Tetrodotoxin concentrations and masses among New Zealand populations of *Pleurobranchaea maculata*. Surveys were also undertaken at Narrow Neck Beach on 26/1/2011, 9/2/2011, 24/3/2011 and 13/4/2011 but No *P. maculata* were observed.

Site	Depth (m)	Sampling Date	*n*	Tetrodotoxin (mg kg^−1^)				*Pleurobranchaea maculata* Mass (g)			
				Ave	Min.	Max.	Std. Error	Ave	Min.	Max.	Std. Error
Narrow Neck Beach	3–6	14/06/2010	10	181.9	9.9	506.0	48.4	7.4	1.3	12.2	0.9
	3–6	12/07/2010	10	85.7	15.2	150.2	15.3	14.3	5.6	20.8	1.5
	3–6	31/08/2010	9	52.6	10.3	120.5	12.1	20.7	14.0	42.7	2.9
	3–6	11/11/2010	9	21.7	1.7	83.1	8.9	20.7	12.9	33.4	2.4
	3–6	16/12/2010	10	17.5	1.1	48.4	5.4	15.5	7.8	25.7	1.7
Illiomama Rock	6	14/06/2010	10	368.7	22.7	1414.0	146.6	6.3	3.6	9.7	0.6
Bucklands Beach	3–6	11/11/2010	10	28.8	1.5	39.4	12.5	30.8	20.4	39.5	2.1
Tasman Bay	20	05/11/2010	20	0.0	0.0	0.0	-	50.4	24.0	89.5	4.9
Urquharts Bay	3	28/08/2010	10	16.8	1.4	30.4	2.9	23.9	13.1	31.1	1.7
Pilot Bay	3–5	08/06/2011	8	89.6	29.7	205.6	19.7	11.6	8.7	16.3	1.1
Kaikoura	290	18/11/2011	1	0.0	-	-	-	17.5	-	-	-
Cornwallis Beach	3	02/09/2010	1	130.0	-	-	-	12.9	-	-	-
Kau Bay	14	01/08/2010	1	2.2	-	-	-	1.5	-	-	-
Wilson Bay	6	15/12/2010	1	0.1	-	-	-	7.2	-	-	-

**Table 2:** Pairwise *post hoc* comparisons (Tukey HSD test) of mean tetrodotoxin concentrations among New Zealand populations of *Pleurobranchaea maculata*. Bolded values represent statistically significant differences (*p* < 0.05).

	Urquharts Bay	Tasman Bay	Pilot Bay	Illiomama Rock	Bucklands Beach	Narrow Neck
Urquharts Bay		**0.0001**	**0.0072**	**0.0001**	1.0000	0.9981
Tasman Bay	**0.0001**		**0.0001**	**0.0001**	**0.0001**	**0.0001**
Pilot Bay	**0.0072**	**0.0001**		0.4334	**0.0064**	**0.0022**
Illiomama Rock	**0.0001**	**0.0001**	0.4334		1.0000	**0.0001**
Bucklands Beach	1.0000	**0.0001**	**0.0064**	**0.0001**		0.9981
Narrow Neck	0.9981	**0.0001**	**0.0022**	**0.0001**	0.9981	

### 2.2. Temporal Variation in Tetrodotoxin Concentrations in *Pleurobranchaea maculata* from Narrow Neck Beach

All *P. maculata* collected from Narrow Neck Beach contained TTX. However, TTX concentrations in the *P. maculata* sampled declined over time (Table 1), ranging from an average of 184 mg kg^−1^ in June 2010 to 17.5 mg kg^−1^ by December 2010. These differences were found to be statistically significant (*F* = 8.3764, *df* = 4, *p* < 0.0001). Tukey pairwise comparisons revealed samples collected in June, July and August were not significantly different, but samples collected in November and December differed significantly from the June and July samples (Tukey HSD, MS 1.22, *df* = 43, *p* < 0.05). The August, November and December samples were not significantly different (Tukey HSD, MS 1.22, *df* = 43, *p* > 0.05). Within sampling periods there was considerable variability in TTX concentrations from one individual to the next with a 10 (12 July 2010) to 51 (14 June 2010) fold difference in the minimum and maximum TTX concentrations (Table 1). No *P. maculata* were found at the Narrow Neck Beach site after December 2010, despite four extensive surveys (Table 1).

**Figure 2:**
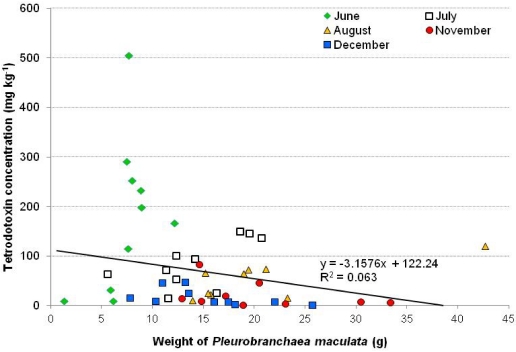
Tetrodotoxin concentrations in individual *Pleurobranchaea maculata* relative to total mass. *Pleurobranchaea maculata* were collected from Narrow Neck Beach (Auckland, New Zealand) between 14 June 2010 to 16 December 2010.

There was no relationship between concentrations of TTX in *P. maculata* and their mass (*R*^2^ = 0.063; Figure 2). The highest levels (506 mg kg^−1^), were measured in an individual that weighed 7.7 g, but low levels (10 mg kg^−1^) were measured in a *P. maculata* of similar mass (6.3 g) collected on the same date (Figure 2).

### 2.3. Rearing of a Second Generation *Pleurobranchaea maculata* in Captivity and Associated Tetrodotoxin

The larvae collected at 5 and 10 days post-hatching were positive for TTX. Only one larva was successfully reared to full maturation. No TTX was detected in this second generation *P. maculata*.

### 2.4. Cytochrome Oxidase Subunit 1 Gene Analysis of Narrow Neck Beach and Tasman Bay *Pleurobranchaea maculata*

Segments (659 bp) of the CO1 gene were successfully amplified from the Narrow Neck and Tasman Bay *P. maculata* samples. Among the four sequences there were only two nucleotides that differed. One of these differences occurred between the two Narrow Neck Beach samples and the other was a consistent difference in the sequences between sampling sites. The sequences only had low sequence homology to other organisms in GenBank; 82% to *Pleurobranchus peroni* (DQ237993), and 81% to *Opisthobranchia* sp. (HM425491) and *Berthella plumula* (AY345025).

## 3. Discussion

Significant variability in TTX concentrations occurred between *P. maculata* populations. No TTX was detected in the *P. maculata* population from Tasman Bay, Nelson and an individual from Kaikoura, while from Wellington to Whangarei populations showed a wide range of TTX levels. Historically there has been confusion as to whether there are multiple *Pleurobranchaea* species in New Zealand [20]. Additionally in this study morphological differences, e.g., mantel pigmentation, were observed between the Tasman Bay and other populations. To eliminate the possibility that the Tasman Bay population was a different species from the North Island population’s analysis of a segment of the CO1 gene was undertaken. This showed <1% sequence variability between the Tasman Bay and Narrow Neck populations providing evidence that these populations are the same species. Variability in TTX levels across populations has been documented elsewhere. For example, populations of the newt *T. granulosa* from north western USA were found to have highly variable levels of TTX, ranging from 0.007 (±0.004) mg g^−1^ to 1.752 (±0.110) mg g^−1^, while those collected further north around Vancouver Island, Canada, did not contain TTX [18]. In *T. granulosa*, variability in TTX levels is attributed to a coevolutionary interaction with the common garter snake *T. sirtalis.* In an apparent coevolutionary arms race, *T. sirtalis* is believed to have evolved TTX resistance in parallel with the toxicity level of *T. granulosa* [19]. Such a tight predator-prey relationship is an unlikely explanation for variability among *P. maculata* populations. In our studies we observed TTX-containing *P. maculata* in a diverse range of habitats ranging from sandy sediment to rocky reefs, where they would be exposed to predation from multiple marine organisms including fish and octopus. Geographic variability among marine TTX-containing organisms occurs in multiple species including: puffer fish [22,23], the horse-shoe crab *Carcinoscorpius rotundicauda* [24,25,26] and the starfish *Astropecten polyacanthus* [27]. Although definitive reasons for these variations are unknown the most likely explanation is that these organisms are exposed to TTX through the food chain [28] and that the abundance of this dietary source influences the presence and levels of TTX within an organism. Noguchi *et al.*, [28] provided evidence to support this hypothesis by culturing over 5000 specimens of the puffer fish *Takifugu rubripes* in net-cages and land-based aquaria. These organisms were fed TTX-free diets for between 1–3 years and all livers and other tissues tested negative for TTX. A dietary source of TTX is a plausible explanation for the variation in TTX levels in *P. maculata,* however in-depth benthic surveys of the flora and fauna at sites with high *P. maculata* abundance have only detected trace levels of TTX in a few organisms (e.g., the Sand Dollar *Arachnoides zelandiae* and the crab *Macrophthalmus hirtipes*), all of which are unlikely to be an abundant food source for *P. maculata* [29]. It is possible that if the dietary source was microbial it may not have been detected in these surveys and attempts are currently underway to isolate TTX-producing bacteria from *P. maculata*. Recently latitudinal gradients in marine planktonic bacteria have been demonstrated [30], and it is possible that latitudinal and current-induced gradients may result in differences in bacterial diversity within *P. maculata* populations around New Zealand. While the distances between Kaikoura and Tasman Bay (non-toxic populations) and the Wellington site (toxic individual) are only 175–215 km respectively, there is limited opportunity for oceanic exchange between theses South and North Island sites, with the prevailing oceanic currents flowing east from Tasman Bay and north from Kaikoura [31,32].

In addition to between-population variability there were also significant difference in TTX concentration within toxic *P. maculata* populations, with the most pronounced difference (>60 fold) occurring among individuals collected from Illiomama Rock. Variability in TTX-concentrations among populations has been measured in many TTX-containing organisms. In the most extreme examples both non-toxic and highly toxic individuals are found in a single population e.g., the gastropods *Rapana rapiformis* and *R. venosa venosa* [33] and the horseshoe crab *Carcinoscorpius rotundicauda* [26]. In other species populations tend to be either toxic or non-toxic, but within the TTX-positive populations there is a marked range in toxin levels e.g., *T. granulosa* [34,35]. We considered the possibility that the concentration of TTX may be related to the size of individual *P. maculata* and this may partly explain the variability. If TTX is produced endogenously it would be logical to expect larger *P. maculata* capable of producing more TTX and thus higher concentrations would be associated with heavier individuals. However, no correlation between mass and TTX concentrations was observed (Figure 2). Similar patterns have been noted in other TTX-containing organisms. For example, there was no correlation between toxicity and body mass in specimens collected at the same place and time of the starfishes *Astropecten polyacanthus* and *A. scoparius* [27] or the ribbon worm *Cephalothrix* sp. [36]. Genetic variation among individual may also affect their ability to produce or sequester TTX and thus could possibly explain the lack of relationship between mass and TTX concentrations. 

Over seven months the population of *P. maculata* at Narrow Neck Beach (Auckland, New Zealand) showed a trend of decreasing levels of TTX from an initial peak in June-July to a low in December. In other TTX-containing organisms there are conflicting data regarding seasonal fluctuations. For example, although toxicity varied among samples there were no clear seasonal trends in studies of two gastropods; the lined-moon shell, *Natica* lineata [37] and *Nassarius* *glans* [38]. In contrast, ribbon worms (*Cephalothrix* sp.) collected from November 1998 to March 1999 showed mean toxicities of 2101–3278 MU g^−1^. These decreased in April and March, 1999 (995 MU g^−1^), before increasing sharply to (5843 MU g^−1^) in June [36]. Likewise in their studies of the starfish *Astropecten polyacanthus*, Miyazawa *et al.* [39] found that all specimens collected in autumn and winter werenon-toxic. Recent studies on puffer fish have associated seasonal changes in TTX concentrations with different sexual maturity stages. Sabrah *et al.* [40] studied different sexual maturity stages in the puffer fish (*Lagocephalus sceleratus*) and found highest toxicity levels during the spawning stage of maturity. Most of the specimens in maturity stage II (developing stage) were non-toxic. A similar pattern was observed in female *Takifugu poecilonotus* where liver toxicity was highest in the “ordinary period” (just after spawning) and ovary toxicity was highest during the “maturation period” [41]. Although little is known about the ecology of *P. maculata*, they were most abundant and significant quantities of egg masses were observed during our surveys in June to August [29]. This was also the period where the highest TTX-levels were measured suggesting a link between maturation stage and TTX levels in *P. maculata*. Our previous research and ongoing studies have detected high levels of TTX in *P. maculata* eggs and larvae, and demonstrate TTX levels decrease in consecutive batches of eggs [42,43]. Thus, *P. maculata* may invest their offspring with TTX or inoculate them with TTX-producing bacteria as a defense mechanism.

To investigate whether TTX-producing bacteria are transmitted vertically between generations we attempted to rear *P. maculata* in captivity. The two sub-samples of planktonic larvae tested positive for TTX. Despite more than 20 pediveligers settling and more than 10 metamorphosing into juveniles, only one survived to full maturity and no TTX was detected. Unfortunately with such a limited sample size we cannot draw any definitive conclusions from this experiment. Similar experiments with captive-raised pufferfish [17] and riparian frogs (*Atelopus variusdo*) [44] have also found that second generation cohorts do not possess TTX. In both of these examples the researchers suggest that the lack of TTX in these captivity-reared organisms indicates that TTX may either have a dietary origin or may be dependent on environmental factors, such as microbial symbionts. Gall *et al.* [45] detected TTX in all developmental stages of *T. granulosa*, however, TTX concentrations declined steadily through metamorphosis. In contrast, TTX levels in the blue-ringed octopuses (*Hapalochlaena* spp.) continue to increase during development, suggesting that offspring or their symbionts begin producing TTX independently [46]. Further research tracking TTX levels through all development stages of *P. maculata* is required to understand it role and origin in each life stage.

## 4. Conclusions

In summary, our initial studies on TTX in *P. maculata* show that this organism conforms to the trends observed in other marine TTX-containing organisms. There is significant variability in TTX concentrations within and among populations. TTX concentrations were highest during the egg laying season (June–August) and this, in concert with the detection of high levels of TTX in eggs and early larval stages [42], suggests that TTX may be used for defensive purposes in *P. maculata*. Confounding this hypothesis is the existence of populations that do not contain TTX. It is possible that these populations contain alternative chemical defense mechanisms but this requires further investigation. Our present results suggest that the occurrence of TTX in *P. maculata* may involve symbiotic TTX-producing bacteria, though the possibility that TTX is accumulated through the food chain or produced endogenously cannot be fully excluded.

## 5. Experimental Section

### 5.1. Sampling Sites and Collection

Up to 20 *P. maculata* were collected at 7 sites around New Zealand by divers (Figure 1; Table 1). Water was removed (metal sieve, 1 mm dia., 1 min) and each was frozen (−20 °C) in separate plastic bags before transportation. The individual from Kaikoura (Figure 1; Table 1) was collected using a fish trap (290 m depth) as part of a Museum of New Zealand Te Papa Tongarewa marine surveying programme. The samples from Kau Bay and Cornwallis Beach were opportunistically collected by local divers. At the Narrow Neck Beach site, up to 10 *P. maculata* were collected approximately monthly over a 10-month period (Table 1). 

### 5.2. Rearing of *Pleurobranchaea maculata*

Two *P. maculata* collected from Narrow Neck Beach (Figure 1) on 14 June 2010 were placed in plastic bags containing seawater (300 mL) and transported back to the laboratory in an insulated container. In the laboratory they were maintained in an aquarium (19 L) filled with 11 L of filtered seawater and aerated using a fish tank pump. Twice a week they were fed Greenshell^TM^ mussel (*Perna canaliculus*) and their water exchanged. *Pleurobranchaea maculata* began laying eggs within two weeks. The egg mass used in this study was laid on 16 July 2010. The egg mass was transferred to a 1 L glass beaker containing 500 mL of filtered sea water. The eggs were maintained, and all further rearing undertaken using the following standard conditions: 100 ± 20 µmol photons m^−2^ s^−1^; 12:12 h light:dark; 18 ± 1 °C, without aeration. Larvae hatched within ten days and were transferred to 2 L glass beakers containing 1 L filtered seawater, without aeration. Larvae were fed a 1:1:1 mixture of *Chaetoceros calcitrans*, *Isochrysis galbana* and *Pavlova lutheri* three times weekly and 20 µL of CoralAmino (Brightwell Aquatics) once a week. At 5 and 10 days post-hatching 30 mL subsamples of water containing larvae were collected and centrifuged (3000 × g, 5 min). The pellets were processed and analyzed for TTX as described below. After approximately 2 weeks larvae were transferred to 1 L glass beakers with a 1-week growth of biofilm on the glass surface. Settlement of the pediveligers occurred after approximately 3 weeks. Acquisition of a juvenile morphology occurred gradually over 3 weeks. Juveniles were fed commercial salmon food, and small pieces of Greenshell^TM^ mussel were gradually introduced to their diet as they increased in size. Only one juvenile survived and after approximately six months at a mass of 19.1 g it was frozen, and processed and analyzed for TTX as described below. 

### 5.3. Tetrodotoxin Extraction and Analysis

Each *P. maculata* was homogenized and a sub-sample (2 g) extracted with 18 mL of Milli-Q containing 0.1% v/v acetic acid. The samples were sonicated (20 min), centrifuged (3000 × g, 10 min) and an aliquot of the supernatant analysed for TTX using liquid chromatography-mass spectrometry (LC-MS) as described in McNabb *et al.* [42].

### 5.4. DNA Extraction and CO1 Analysis

DNA was extracted from a small piece of mantle from two *P. maculata* from Narrow Neck Beach and two from Tasman Bay using the E.Z.N.A. Mollusc DNA Kit (Omega, USA) according to the protocol supplied by the manufacturer. PCR reactions were performed in 50 µL volumes with the reaction mixture containing; 25.0 µL of i-Taq 2× PCR master mix (Intron, Gyeonggi-do, Korea), 0.4 μM of each primer (LC01490 and HC02198; [47]) and 10–20 ng of template DNA. The reaction mixture was held at 94 °C for 2 min followed by 35 cycles of 94 °C for 30 s, 45 °C for 30 s, 72 °C for 1 min, with a final extension of 72 °C for 7 min. PCR reactions were run on an iCycler thermal cycler (Biorad, USA). PCR products were visualized on 1% agarose gel and then purified using an AxyPrep PCR cleanup kit (Axygen, CA, USA). Bi-directional sequencing was undertaken using the BigDye Terminator v3.1 Cycle Sequencing Kit (Applied Biosystems, CA, USA). Sequences obtained in this study were deposited in the NCBI GenBank database under accession numbers JN675220-23. These were compared with sequences from the NCBI GenBank database using MegaBlast [48].

### 5.5. Statistical Analysis

Data were log transformed and the Cochrans test for homogeneity was non-significant. One-way ANOVAs were used to compare mean concentrations of TTX from six sites and between each of the six sampling times at the Narrow Neck site. Post-hoc pairwise comparisons of all mean TTX concentrations at six sampling sites and between the six sampling times at the Narrow Neck site were undertaken using the Tukey HSD test function in Statistica 8 [49].
